# Applying Nanoscale Kirkendall Diffusion for Template-Free, Kilogram-Scale Production of SnO_2_ Hollow Nanospheres *via* Spray Drying System

**DOI:** 10.1038/srep23915

**Published:** 2016-04-01

**Authors:** Jung Sang Cho, Hyeon Seok Ju, Yun Chan Kang

**Affiliations:** 1 Department of Materials Science and Engineering, Korea University, Anam-Dong, Seongbuk-Gu, Seoul 136-713, Republic of Korea

## Abstract

A commercially applicable and simple process for the preparation of aggregation-free metal oxide hollow nanospheres is developed by applying nanoscale Kirkendall diffusion to a large-scale spray drying process. The precursor powders prepared by spray drying are transformed into homogeneous metal oxide hollow nanospheres through a simple post-treatment process. Aggregation-free SnO_2_ hollow nanospheres are selected as the first target material for lithium ion storage applications. Amorphous carbon microspheres with uniformly dispersed Sn metal nanopowder are prepared in the first step of the post-treatment process under a reducing atmosphere. The post-treatment of the Sn-C composite powder at 500 °C under an air atmosphere produces carbon- and aggregation-free SnO_2_ hollow nanospheres through nanoscale Kirkendall diffusion. The hollow and filled SnO_2_ nanopowders exhibit different cycling performances, with their discharge capacities after 300 cycles being 643 and 280 mA h g^−1^, respectively, at a current density of 2 A g^−1^. The SnO_2_ hollow nanospheres with high structural stability exhibit superior cycling and rate performances for lithium ion storage compared to the filled ones.

Metal-oxide hollow nanospheres with a high surface area and a sufficiently void internal volume can be used in various applications, including energy storage devices, solar cells, catalysts, sensors, and drug delivery^1^,^2^,^3^,^4^,^5^,^6^,^7^,^8^,^9^. Hollow nanospheres are typically prepared using easily removable organic or inorganic nanopowders as template materials^8^,^9^,^10^,^11^,^12^,^13^. The template nanopowders are uniformly coated with metal precursors by hydrothermal or precipitation methods. The post-treatment process forms a metal oxide layer and eliminates the template nanopowder, producing a metal oxide hollow nanosphere. Monodisperse organic polymer nanopowders, such as polystyrene (PS) and poly(methyl methacrylate) (PMMA), which are mainly prepared by emulsion methods, have been used because they are easily removed by combustion under an oxygen atmosphere. In addition, monodisperse silica nanopowders prepared by a modified Stöber method have been used as inorganic templates because silica can be eliminated with a HF or NaOH solution^6^,^14^,^15^. However, the processes for producing hollow nanospheres using organic and inorganic nanopowders as templates are time consuming and not cost-effective. Moreover, the yield is often on the scale of milligrams to grams, limiting their application for large-scale production of homogeneous metal oxide hollow nanospheres.

In recent years, conversion chemical reactions employing nanoscale Kirkendall diffusion have received significant attention as a method for producing hollow nanospheres^16^,^17^,^18^,^19^,^20^,^21^,^22^. Metal nanopowders formed by a reduction reaction in aqueous media are transformed into hollow nanospheres by nanoscale Kirkendall diffusion; however, that preparation of homogeneous nanopowders is not easily carried out without aggregation. As a result, oxidation of metal nanopowders by the nanoscale Kirkendall diffusion process is typically performed by bubbling oxygen through the aqueous media^16^,^17^. Alternatively, metal nanopowders are oxidized over the carbon-coated Cu grids used for TEM measurements^19^,^20^. Therefore, the development of a simple process for large-scale production of metal oxide hollow nanospheres remains a large challenge if widespread application is to take place.

In this study, a simple and commercially applicable process to produce aggregation-free metal oxide hollow nanospheres has been developed, which uses large-scale spray drying. The precursor powders prepared by the spray drying process are transformed into homogeneous metal oxide hollow nanospheres by a simple two-step post-treatment process. Transition metal oxide hollow nanospheres can be successfully used as anode materials for lithium ion batteries (LIBs) as a result of their short Li-ion diffusion length and good accommodation of volume changes resulting from repeated insertion and extraction of Li^4^,^5^,^6^,^23^,^24^,^25^,^26^,^27^,^28^. Nanostructured tin oxide (SnO_2_) materials, which have a wide band gap of 3.54 eV, are widely applied in various fields, such as gas sensors and catalysts as well as energy storage devices^6^,^29^,^30^,^31^,^32^,^33^,^34^,^35^,^36^,^37^,^38^. In this study, the LIB anode was selected as the first target application for aggregation-free SnO_2_ hollow nanospheres. The detailed formation mechanism of aggregation-free SnO_2_ hollow nanospheres by the nanoscale Kirkendall diffusion process was studied by investigating morphological changes of the spray dried powders during the post-treatment process under reducing and oxidizing atmospheres.

## Results and Discussion

**Figure 1:**
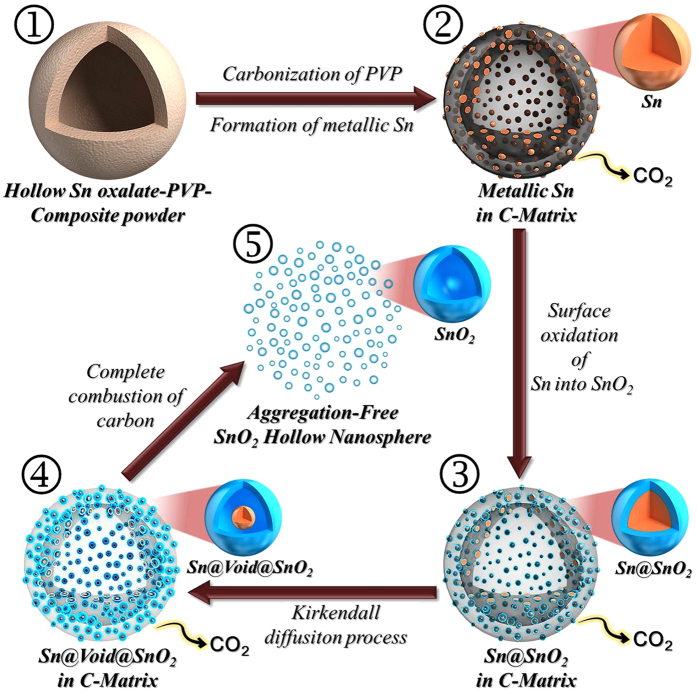
Schematic diagram for the formation mechanism of the aggregation-free SnO_2_ hollow nanospheres.

**Figure 2:**
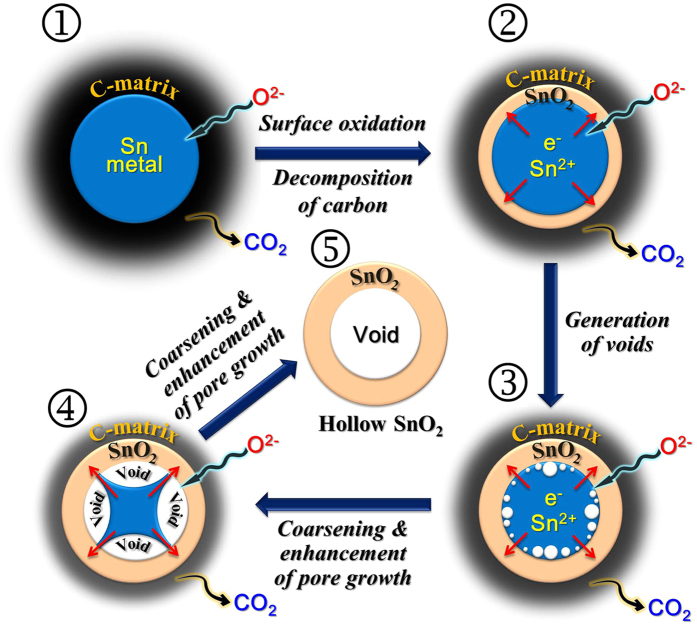
Possible formation mechanism of a hollow SnO_2_ nanosphere by Kirkendall diffusion effect and its chemical conversion process in the surface region of a sphere.

A diagram illustrating the formation of aggregation-free SnO_2_ hollow nanospheres is given in Fig. 1. The Sn oxalate-PVP composite powder prepared by a pilot-scale spray drying process showed particles several tens of micrometers in size (Fig. 1①). Post-treatment of the spray-dried powder at 300 °C under a H_2_/Ar gas mixture produced the Sn-C composite powder (Fig. 1②). Carbonization of PVP during the post-treatment process produced an amorphous carbon matrix. Decomposition of Sn oxalate into tin oxide occurred, with subsequent reduction to Sn metal. The segregation of Sn during reduction, with its low melting temperature, resulted in aggregation-free Sn metal nanopowders. Next, post-treatment of the Sn-C composite powder at 500 °C under an air atmosphere produced carbon-free and aggregation-free SnO_2_ hollow nanospheres through the process of well-known nanoscale Kirkendall diffusion (Fig. 1③). The Kirkendall effect, a vacancy flux and subsequent void formation process resulting from diffusivity differences at inorganic interfaces, was described in detail in Fig. 2. The Kirkendall effect results in the formation of a thin SnO_2_ layer on the Sn metal surface, followed by simultaneous outward diffusion of Sn cations through the oxide layer and inward diffusion of oxygen into the nanospheres, creating an intermediate Sn@SnO_2_ core–shell structure (Fig. 2④). Sn cations diffused outward more quickly than oxygen diffused inward, which is consistent with the larger ionic radius of oxygen anions (140 pm) than Sn cations (Sn^2+^ is 93 pm, Sn^4+^ is 69 pm). Accordingly, Kirkendall voids were generated near the Sn/SnO_2_ interface during vacancy-assisted exchange of the material *via* bulk interdiffusion (Fig. 2⑤), which gave rise to coarsening and enhancement of pore growth in the spheres (Fig. 2⑥). Eventually, both complete conversion of Sn metal into SnO_2_ by Kirkendall-type diffusion and complete combustion of the amorphous carbon material surrounding the Sn metal spheres resulted in the carbon-free and aggregation-free SnO_2_ hollow nanospheres (Fig. 2⑤).

The formation mechanism of the carbon-free and aggregation-free SnO_2_ hollow nanospheres was investigated on the basis of morphology changes induced by post-treatment in the Sn oxalate-PVP composite powder. The characteristics of the precursor powders produced by the spray drying process are shown in Figure S3. The x-ray diffraction (XRD) pattern of the precursor powders reveals the broad crystalline peaks of the tetragonal SnO_2_ phase. The thermogravimetric (TG) curve shown in Figure S3b shows a three-step weight loss of the precursor powders below 500 °C. The distinct weight losses at temperatures around 170, 260 and 380 °C were attributed to decomposition of tin oxalate, carbonization of PVP, and combustion of the carbon component, respectively. The decomposition of some amount of tin oxalate into SnO_2_ occurred in the spray drying process, as shown by XRD and TG analysis. The precursor powders showed the typical collapsed structure of hollow powders. The formation of a gas-impermeable layer during an early drying stage of the droplets resulted in hollow powders, with further gas evolution by water evaporation expanding the balloon–like structures. The explosion of expanded powder resulted in collapsed structures with holes, as shown by arrows in Figure S3c.

**Figure 3:**
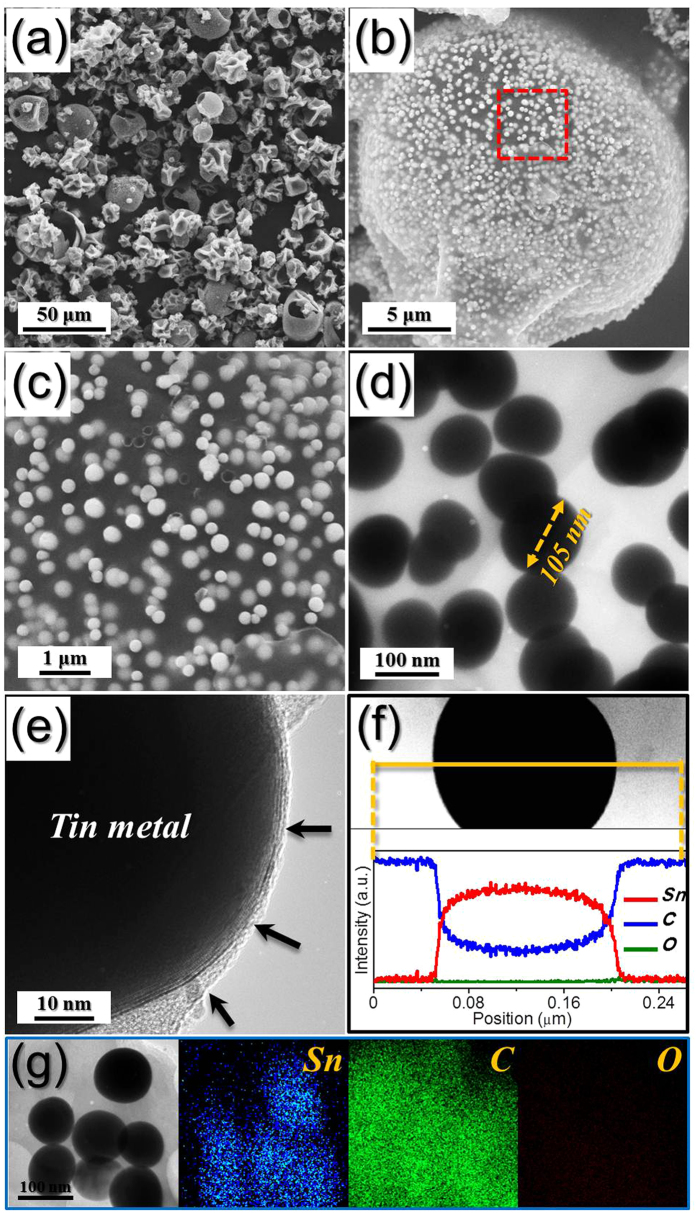
Morphologies, line profiling analysis, and elemental mapping images of the Sn-C composite powders obtained by reduction of the spray dried precursor powders at 300 °C under 10% H_2_/Ar gas: (**a–c**) SEM images, (**d,e**) TEM images, (**f**) line profiling analysis, and (**g**) elemental mapping images.

The characteristics of the Sn-C composite powders obtained by reduction of the spray dried precursor powders are shown in Fig. 3 and Figure S4a. The XRD pattern shown in Figure S4a revealed the marked crystalline peaks of the metallic Sn phase. Complete decomposition of tin oxalate into tin oxide and subsequent reduction to metallic Sn occurred during this stage. The low-resolution scanning electron microscope (SEM) image shown in Fig. 3a reveals a similar morphology to that of the spray dried precursor powder. However, the high-resolution SEM and TEM images in Fig. 3b–d reveal a unique morphology of the powders prepared by the reduction process. Ultrafine nanopowders with a narrow size distribution were uniformly distributed over the transparent powder. The amorphous carbon formed by carbonization of PVP appears as a transparent matrix supporting the nanopowders in the SEM and TEM images. The early stage of the reduction process formed the homogeneous amorphous carbon-tin oxide composite powder as an intermediate product. Reduction of tin oxide into metallic Sn changed the morphology of the powders, and segregation of Sn metal with its low melting temperature occurred within the amorphous carbon matrix to form aggregation-free Sn nanopowders. The Sn nanopowder located close to the surface of the Sn-C composite powder also shows a carbon layer, indicated by an arrow in Fig. 3e. The line profiling and elemental mapping images revealed pure Sn metal nanopowder embedded within the amorphous carbon matrix (Fig. 3f,g). The mean particle size of the Sn metal nanopowders measured from the SEM images was 95 nm. The TG curve of the Sn-C composite powders in Figure S5a revealed a slight weight increase beginning at 150 °C and distinct weight loss between 350 and 380 °C. Weight increase from oxidation of metallic Sn nanopowder was reduced by combustion of amorphous carbon. The amorphous carbon content of the Sn-C composite powders estimated from the TG analysis was 50 wt%.

**Figure 4:**
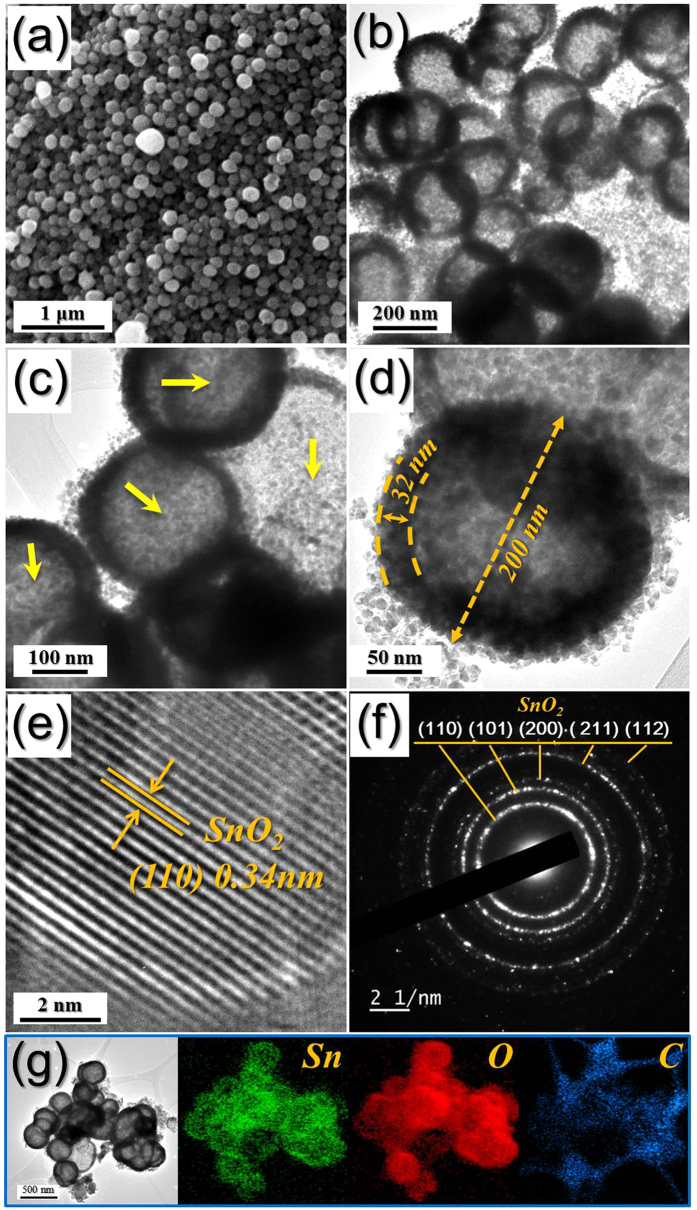
Morphologies, SAED pattern, and elemental mapping images of the SnO_2_ hollow nanospheres obtained by oxidation of reduced Sn-C composite powders at 500 °C under air: (**a**) SEM, (**b–d**) TEM images, (**e**) HR-TEM image, (**f**) SAED pattern, and (**g**) elemental mapping images.

The characteristics of the tin oxide powders obtained by oxidation of the Sn-C composite powders at 500 °C are shown in Fig. 4 and Figure S4b. The XRD pattern shown in Figure S4b reveals a mixed crystal structure of tetragonal and orthorhombic SnO_2_ phases; complete oxidation of the metallic Sn nanopowders into SnO_2_ occurred during the process. Morphology of the powders has changed drastically with oxidation. The Sn-C composite powder with particles several tens of micrometers in size has changed into SnO_2_ nanopowder with nanometer-sized particles, as a result of combustion of the amorphous carbon matrix. The TEM images revealed the hollow structure of the SnO_2_ nanopowders. The Sn nanopowder, which had a dense structure, was transformed into hollow SnO_2_ nanospheres by the well-known nanoscale Kirkendall diffusion process elucidated in Fig. 4. A clear void space was observed inside the SnO_2_ nanopowders as indicated by arrows in Fig. 4c. The shell thickness and diameter of the SnO_2_ nanospheres shown in Fig. 4d were 32 and 200 nm, respectively. Ultrafine SnO_2_ nanocrystals below 8 nm constituted the hollow thin shell. The enlarged TEM image in Fig. 4e shows clear lattice fringes separated by 0.34 nm, corresponding to the (110) lattice plane of tetragonal SnO_2_. The selected area electron diffraction (SAED) pattern shown in Fig. 4f reveals the highly crystalline structure of the SnO_2_ hollow nanospheres. The elemental mapping images and TG curve shown in Fig. 4g and Figure S5b, respectively, show a trace amount of the carbon component in the SnO_2_ hollow nanospheres. Further evidence for the oxidation of metallic Sn into SnO_2_ during the post-treatment in air is provided by the XPS analysis in Figure S6. Deconvolution of the XPS Sn 3d peaks at binding energies of 487.7 eV (Sn 3d_5/2_) and 496.0 eV (Sn 3d_3/2_) shows a SnO_2_ layer in addition to the metallic Sn nanopowder (Figure S6a); this oxide resulted from partial surface oxidation of Sn nanocrystals by exposure to air. However, the XPS spectrum of the SnO_2_ hollow nanospheres (Figure S6b) shows Sn peaks for only the oxide form, with binding energies of 487.0 eV (Sn 3d_5/2_) and 495.5 eV (Sn 3d_3/2_). This provides evidence that the Sn-C composite powder was completely transformed into aggregation-free SnO_2_ hollow nanospheres by a simple oxidation process.

For lithium ion storage devices, electrochemical properties were compared between SnO_2_ hollow nanospheres and SnO_2_ nanopowders with a filled structure, as prepared by one-pot flame spray pyrolysis. The nanopowder formation mechanisms in flame spray pyrolysis have been described in our previous publications^39^. The drying and decomposition of a droplet inside the diffusion flame formed a micron-sized SnO_2_ powder. Complete evaporation of SnO_2_ powder in the high-temperature diffusion flame formed the vapors of SnO_2_. The SnO_2_ nanopowders were formed by nucleation and growth mechanisms from the SnO_2_ vapors. The TEM images shown in Figure S7b–d reveal the filled structure of highly crystalline SnO_2_ nanopowders. The mean crystallite and particle sizes of the SnO_2_ nanopowders were 27 and 34 nm, respectively, measured from the XRD pattern and TEM image. The Brunauer-Emmett-Teller (BET) surface area of the SnO_2_ nanopowders was 22 m^2^ g^−1^ for both the filled and hollow structures (Figure S8).

The cyclic voltammogram (CV) curves of the two samples for the first 5 cycles at a scan rate of 0.07 mV s^−1^ are shown in Figure S9. The CV curves of the two samples had similar shapes. However, the relative intensity of the reduction peak observed at around 0.8 V in Figure S9a was lower than in Figure S9b. In the first cathodic step, the apparent reduction peak observed at around 0.8 V was mainly associated with the formation of metallic Sn nanograins and amorphous Li_2_O through reduction of SnO_2_^38^,^40^. The ultrafine crystallite size of the SnO_2_ hollow nanospheres broadens the reduction peak observed at around 0.8 V. The broad reduction and oxidation peaks at around 0.2 and 0.5 V, which were attributed to the alloying and de-alloying reactions of metallic Sn with lithium, respectively, were observed in the two samples from the second cycle onward^32^,^33^,^34^,^35^. The good overlapping of the CV curves from the second cycle onward revealed good reversibility of the electrochemical reactions during the first 5 cycles in the two samples.

**Figure 5:**
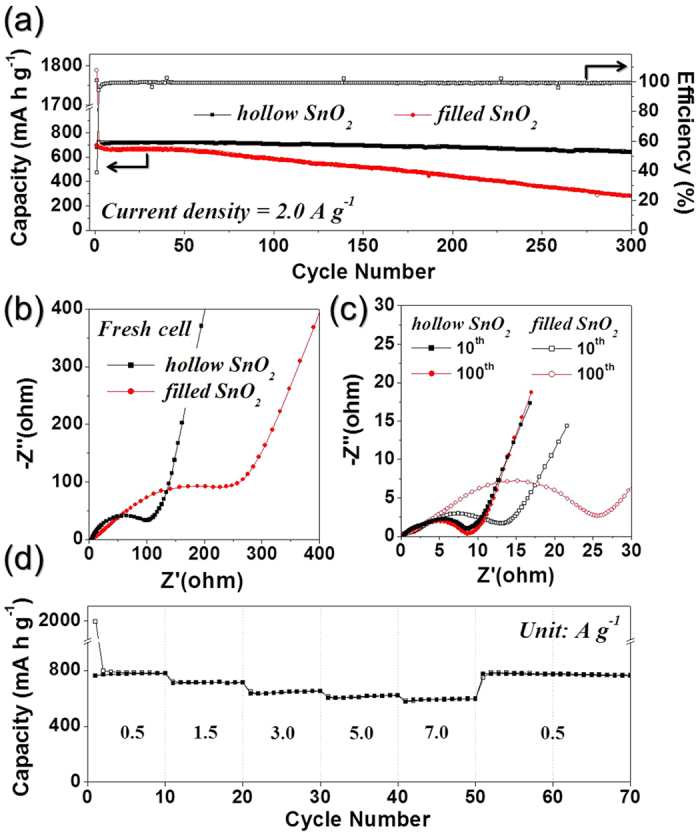
Electrochemical properties of the SnO_2_ hollow nanospheres formed by applying nanoscale Kirkendall diffusion process and filled-structured SnO_2_ nanoparticles formed by conventional flame spray pyrolysis process: (**a**) Cycling performances at a constant current density of 2.0 A g^−1^ and Coulombic efficiencies of the SnO_2_ hollow nanospheres, (**b**) Nyquist impedance plots before cycling, (**c**) Nyquist impedance plots after cycling, and (d) rate performance of the SnO_2_ hollow nanospheres.

The discharge and charge curves of SnO_2_ nanopowders at a constant current density of 2.0 A g^−1^ are shown in Figure S10. The SnO_2_ nanopowders exhibited similarly shaped initial discharge and charge curves irrespective of their morphologies. The clear plateaus at around 0.80 and 0.73 V, which were attributed to the formation of metallic Sn nanograins and amorphous Li_2_O through reduction of SnO_2_, were observed in the initial discharge curves of the hollow and filled SnO_2_ nanopowders, respectively. The initial discharge and charge capacities of the SnO_2_ hollow nanospheres were 1762 and 680 mA h g^−1^, respectively, and its corresponding Coulombic efficiency was 39%. The SnO_2_ nanopowders with filled structure had similar discharge and charge capacities to the hollow nanospheres. However, the two types of SnO_2_ nanopowders had different cycling performances as shown in Fig. 5a. The discharge capacities of the SnO_2_ hollow and filled nanopowders after 300 cycles were 643 and 280 mA h g^−1^, respectively. The hollow SnO_2_ nanopowders could accommodate the large volume variation during repeated lithium insertion and extraction and decrease Li^+^ diffusion length, thus leading to improved cycling stability even at the high current density of 2 A g^−1^.

Electrochemical impedance spectroscopy (EIS) measurements were carried out to explain the superior cycling performance of the SnO_2_ hollow nanospheres compared to the filled nanopowders. The Nyquist impedance plots of the two samples obtained before and after cycling under a fully charged state are shown in Fig. 5b,c. The medium-frequency semicircles in the Nyquist plots of the electrode were assigned to the charge-transfer resistance (*R*_ct_)^31^,^41^,^42^. The SnO_2_ hollow nanospheres with ultrafine crystallite size had a lower charge transfer resistance than the filled nanopowders before cycling (Fig. 5b). The charge transfer resistances of the two samples decreased strictly after the first cycle as a result of formation of ultrafine nanocrystals during the first discharging and charging process. The low charge transfer resistance of the SnO_2_ hollow nanospheres remained constant even after 100 cycles (Fig. 5c); however, the charge transfer resistance of the electrode with SnO_2_ filled nanopowders increased with cycle number, as a result of structural destruction of the SnO_2_ filled nanopowders during cycling. In contrast, the hollow structure of the SnO_2_ nanospheres accommodated the large volume change during repeated lithium insertion and extraction. The high structural stability of SnO_2_ hollow nanospheres during cycling improved their cycling performance even at the high current density of 2 A g^−1^. The rate performance of SnO_2_ hollow nanospheres is shown in Fig. 5d, with the current density increasing stepwise from 0.5 A g^−1^ to 7.0 A g^−1^ with 10 cycles performed at each step. The final rate capacities of the SnO_2_ hollow nanospheres were 780, 714, 653, 621 and 597 mA h g^−1^ at current densities of 0.5, 1.5, 3.0, 5.0 and 7.0 A g^−1^, respectively. The discharge capacity of the SnO_2_ hollow nanospheres recovered well to 783 mA h g^−1^, when the current density was returned to 0.5 A g^−1^ after the 50 cycle test sequence.

The morphologies of the SnO_2_ nanospheres formed by applying nanoscale Kirkendall diffusion process and filled-structured SnO_2_ nanopowders prepared by flame spray pyrolysis process obtained after 300 cycles are shown in Figure S11. The hollow SnO_2_ nanospheres formed by applying nanoscale Kirkendall diffusion process maintained their morphologies quite well even after repeated lithium insertion and desertion processes as shown by TEM images in Figure S11a. However, the filled structured SnO_2_ nanopowders prepared by flame spray pyrolysis process were broken into several pieces and aggregated after repeated cycling (Figure S11b).

**Figure 6:**
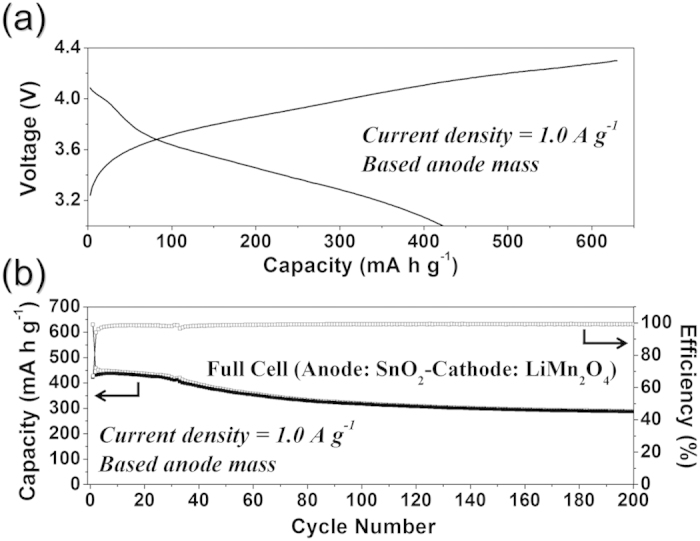
Electrochemical properties of a full cell of anode (hollow SnO_2_ nanospheres)/cathode (LiMn_2_O_4_): (**a**) charge–discharge curves at a current density of 1.0 A g^−1^ and (**b**) cycling performance at a current density at 1.0 A g^−1^ based on the anode (hollow SnO_2_ nanospheres) mass.

To confirm the possibility of the hollow SnO_2_ nano spheres for commercial application, the hollow SnO_2_ nanospheres anode was prelithiated and combined with a high voltage LiMn_2_O_4_ cathode to construct a full Li-ion cell. Yolk–shell structured LiMn_2_O_4_ powders were prepared as a cathode active material by spray pyrolysis process^43^,^44^. The morphologies and phase of the yolk–shell structured LiMn_2_O_4_ powders prepared by spray pyrolysis are shown in Figure S12. The electrochemical performances of the yolk–shell structured LiMn_2_O_4_ powders were shown in Figure S13. The charge and discharge curves and cycling performance of hollow SnO_2_-nanospheres/LiMn_2_O_4_ yolk–shell full cells with a cut-off voltage range of 3.0–4.3 V are shown in Fig. 6. As shown in Fig. 6a, these materials can exhibit charge and discharge capacities of about 630 and 423 mA h g^−1^, respectively, at the first cycle at a current density of 1.0 A g^−1^, based on the mass of hollow SnO_2_-nanospheres anode. In Fig. 6b, the Coulombic efficiency of the cell in the initial cycle was 67%, and it increased quickly to close to an average value of 99% in the following cycles. The discharge and charge capacities of the cell after 200 cycles were 289 and 287 mA h g^−1^, respectively.

## Conclusions

In this study, a simple and commercially viable process for large scale production of aggregation-free metal oxide hollow nanospheres has been described. Application of the nanoscale Kirkendall diffusion process in a large-scale spray drying process enabled the preparation of metal oxide hollow nanospheres. The key idea was the preparation of an amorphous carbon matrix with a uniform dispersion of metal nanopowders as an intermediate product. Metal nanopowders were transformed into metal oxide hollow nanopowders by the nanoscale Kirkendall diffusion process. The amorphous carbon matrix enabled the formation of aggregation-free metal oxide hollow nanospheres. The aggregation-free SnO_2_ hollow nanospheres had superior electrochemical properties for lithium ion storage compared to the SnO_2_ nanopowders with filled structure. The simple process applied in this study could be applied in the preparation of metal oxide hollow nanospheres with various compositions for numerous applications, including energy storage devices.

## Materials and Methods

### Sample preparation

Aggregation-free SnO_2_ nanoparticles with a hollow structure were prepared using a commercial spray-drying system (Figure S1), followed by a simple two step heat-treatment. Spray solution for the synthesis of tin(II) oxalate- polyvinylpyrrolidone (PVP) composite precursor powders was prepared by dissolving 0.1 M tin(II) oxalate (Sn(Oct)_2_, 99.9%, Aldrich) and 15 g PVP [(C_6_H_9_NO)_n_, Mw-1,300,000, Aldrich] in 1 L of distilled water. The prepared spray solution was pumped by an atomizing device (20 mL min^−1^) with a two-fluid nozzle operated at a pressure of 0.2 bar, which generated numerous droplets in a stream of hot air. The spray-dried powder was separated from the humid air centrifugally in a cyclone system. Temperatures at the inlet and outlet of the spray dryer were maintained at 300 °C and 130 °C, respectively. To produce SnO_2_ hollow nanospheres, the Sn(Oct)_2_-PVP composite powders were post-treated at 300 °C in a 10% H_2_/Ar gas mixture for 3 h and subsequently held at 500 °C in air for 5 h. For comparison to the hollow nanospheres, SnO_2_ nanoparticles with a filled structure were prepared from a 0.1 M tin oxalate spray solution without PVP, using a flame spray pyrolysis system (Figure S2) consisting of a droplet generator, flame nozzle, powder collector, and blower. A 1.7 MHz ultrasonic spray generator with 6 resonators was used to generate droplets, which were carried into a high-temperature diffusion flame by carrier gas (oxygen). Propane and oxygen were the fuel and oxidizer, respectively, for the diffusion flame. The flow rates of fuel, oxidizer, and carrier gas were 5, 40 and 5 L min^−1^, respectively.

### Characterization

Microstructures of the prepared powders were observed by field-emission scanning electron microscopy (FE-SEM, Hitachi, S-4800) and field-emission transmission electron microscopy (FE-TEM, JEOL, JEM-2100F). Crystal phases were identified by X-ray diffractometry (XRD, X’Pert PRO MPD), using Cu K_α_ radiation (λ = 1.5418 Å) at the Korea Basic Science Institute (Daegu). X-ray photoelectron spectroscopy (XPS, Thermo Scientific K-Alpha) with focused monochromatic Al K_α_ radiation, operating at 12 kV and 20 mA, was used to analyze specimen composition. Surface areas of the aggregation-free SnO_2_ hollow nanospheres were measured by the Brunauer–Emmett–Teller (BET) method, using N_2_ as adsorbate gas. Thermogravimetric analysis was performed (Pyris 1 TGA, Perkin Elmer) within the temperature range 25–650 °C at a heating rate of 10 °C min^−1^ under a static air atmosphere. An image analyzer (ImageJ, NIH) was used to determine particle size of the nanospheres.

### Electrochemical measurements

Electrochemical properties of the aggregation-free SnO_2_ hollow nanospheres were analyzed by constructing a 2032-type coin cell. The anode was prepared by mixing the active material, carbon black, and sodium carboxymethyl cellulose (CMC) in a mass ratio of 7:2:1. Li metal and microporous polypropylene film were used as the counter electrode and separator, respectively. The electrolyte was 1 M LiPF_6_ dissolved in a mixture of fluoroethylene carbonate and dimethyl carbonate (FEC/DMC; 1:1 v/v). The discharge/charge characteristics of the samples were investigated by cycling in the 0.001–1.0 V potential range at various current densities. Cyclic voltammograms were measured at a scan rate of 0.07 mV s^−1^. The negative electrode using SnO_2_ nanoparticles was of dimensions 1 cm × 1 cm and the mass loading was approximately 1.2 mg cm^−2^. The cathode was prepared by mixing the active material (yolk–shell structured LiMn_2_O_4_), carbon black, and sodium carboxymethyl cellulose (CMC) in a weight ratio of 8:1:1. For full cell assembly, the LiMn_2_O_4_ yolk–shell electrode with a loading mass of 3 mg cm^−2^ was used as a cathode, whereas the anode mass loading was kept at 0.4 mg cm^−2^. For the full cell, the electrolyte was 1 M LiPF_6_ dissolved in a mixture of ethylene carbonate/diethyl carbonate (EC/DEC;1:1 v/v). The electrochemical properties of the 2032-type coin full cells were examined at 1.0 A g^−1^ in voltage windows between 3.0 and 4.3 V. The electrode capacity was calculated according to the weight of the anode materials.

## Additional Information

**How to cite this article**: Cho, J. S. *et al*. Applying Nanoscale Kirkendall Diffusion for Template-Free, Kilogram-Scale Production of SnO_2_ Hollow Nanospheres *via* Spray Drying System. *Sci. Rep.* **6**, 23915; doi: 10.1038/srep23915 (2016).

## Supplementary Material

Supplementary Information
