# Supplementary material for: Applying Nanoscale Kirkendall Diffusion for Template-Free, Kilogram-Scale Production of SnO2 Hollow Nanospheres via Spray Drying System
Source: Sci Rep. 2016 Apr 1;6:23915. doi: 10.1038/srep23915 (PMC4817047; doi:10.1038/srep23915)
Supplement: Supplementary Information [file srep23915-s1.pdf]

Supporting Information

Applying Nanoscale Kirkendall Diffusion for Template-Free, Kilogram-Scale Production of SnO_2 Hollow Nanospheres *via* Spray Drying System

*Jung Sang Cho, Hyeon Seok Ju, and Yun Chan Kang**

J.S. Cho, H.S. Ju, and Prof. Y.C. Kang
Department of Materials Science and Engineering
Korea University
Anam-Dong, Seongbuk-Gu
Seoul 136-713, Republic of Korea
E-mail: yckang@korea.ac.kr

Keywords: Kirkendall diffusion, hollow nanopowders, tin oxide, lithium ion batteries, spray drying

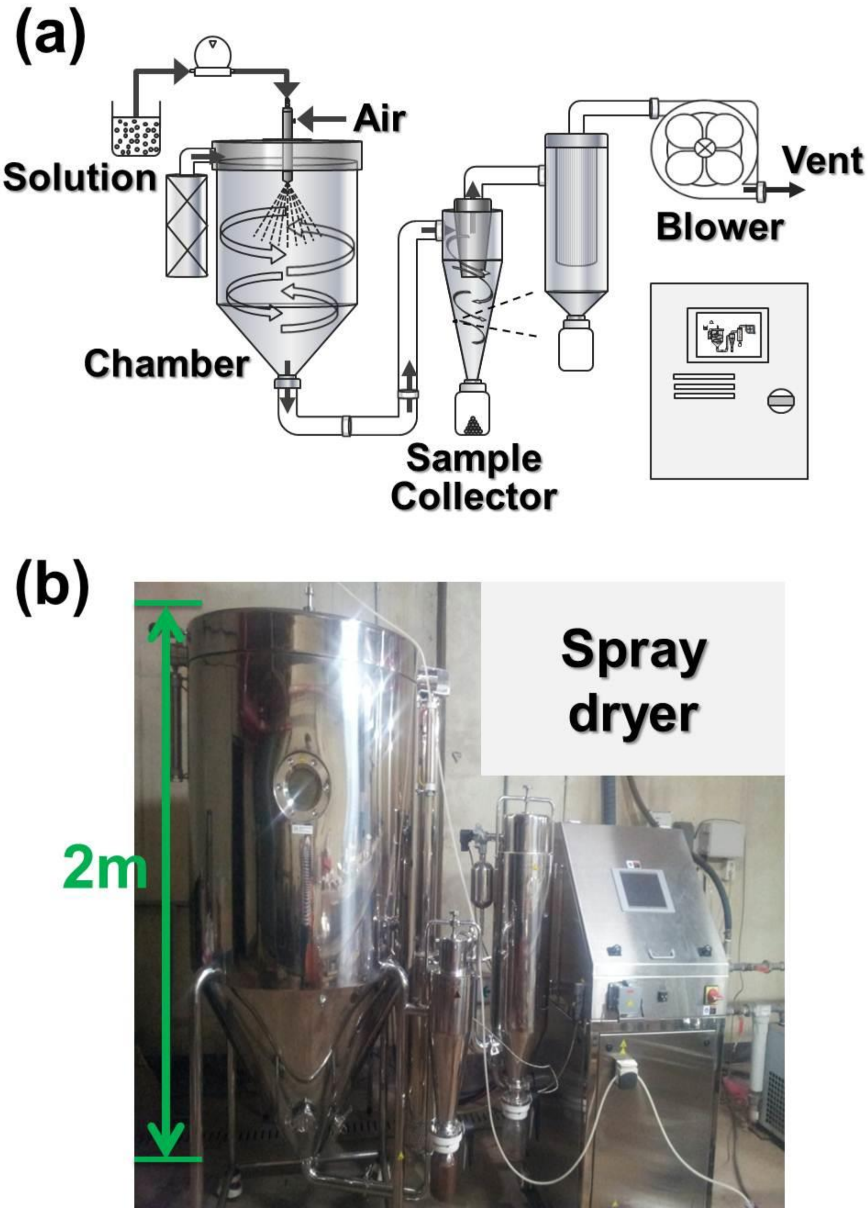

Figure S1 Schematic diagram and digital photo of the commercial spray drying system applied in the preparation of precursor powders.

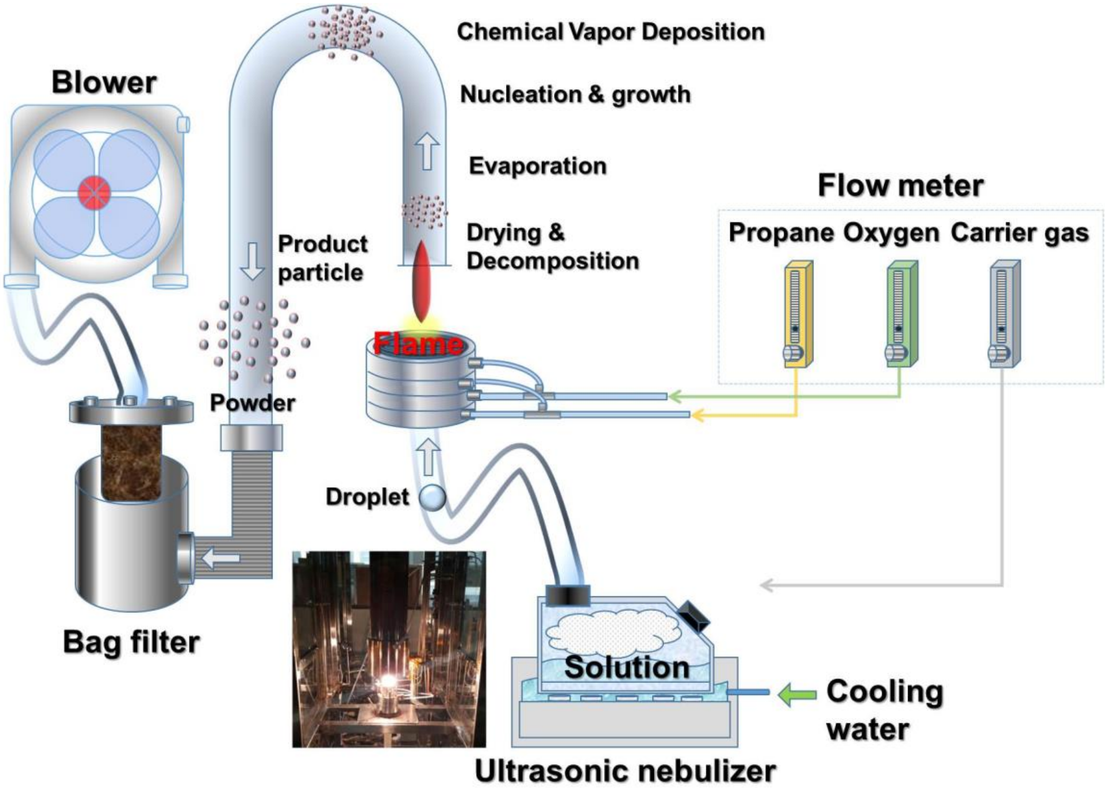

Figure S2 Schematic diagram and digital photo of the flame spray pyrolysis system applied in the preparation of SnO_2 nanoparticles with filled structure.

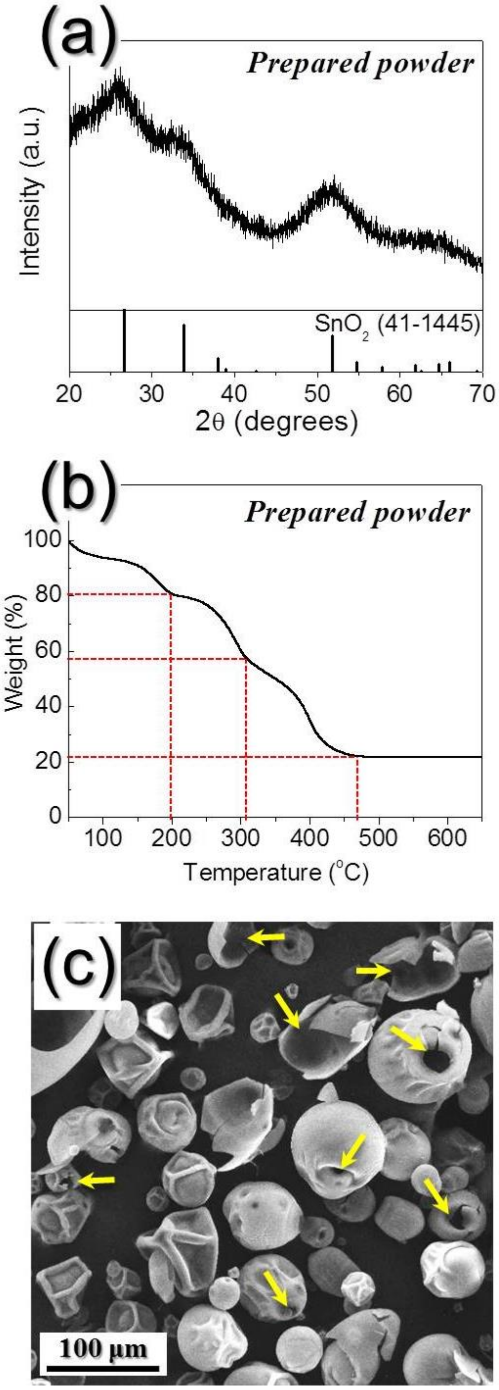

Figure S3 (a) XRD pattern, (b) TG curve, and (c) SEM image of the precursor powders directly obtained by spray drying process.

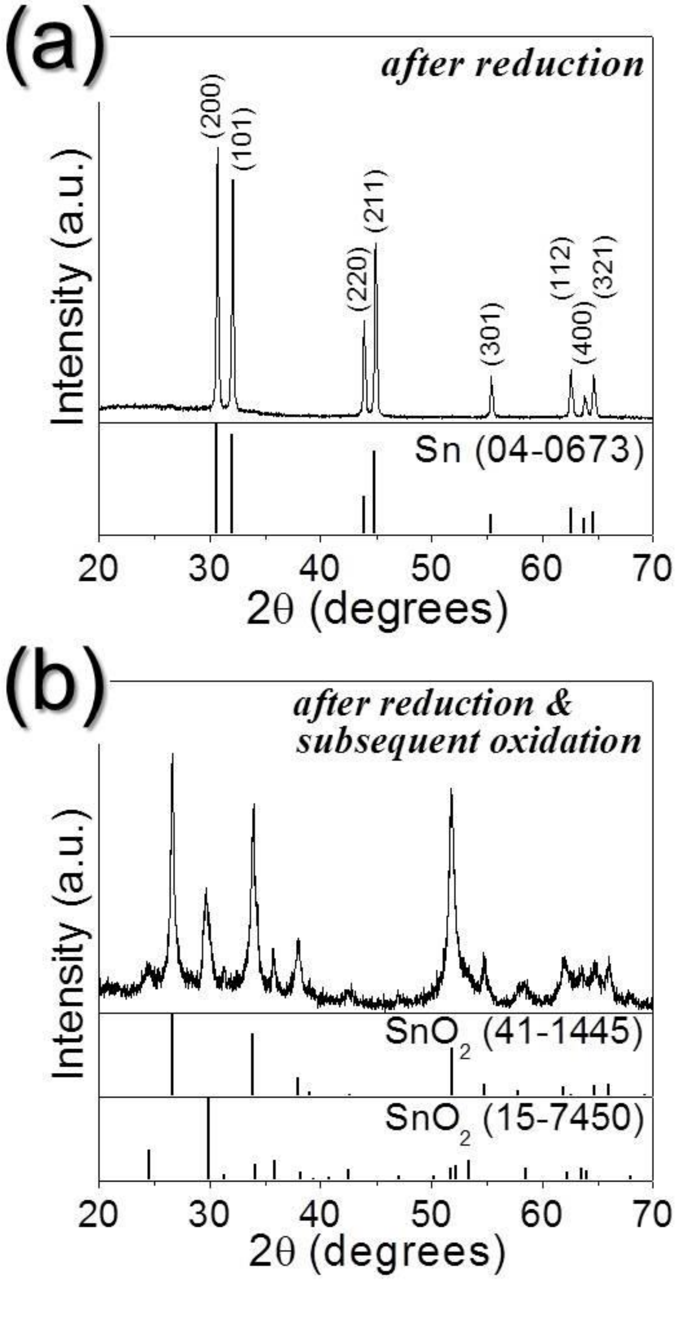

Figure S4 XRD patterns of the (a) Sn-C composite powders obtained by reduction process at 300 ^{o}C under 10% H_2/Ar gas and (b) SnO_2 nanopowders with hollow structure obtained by oxidation of reduced Sn-C composite nanofibers at 500 ^{o}C under air.

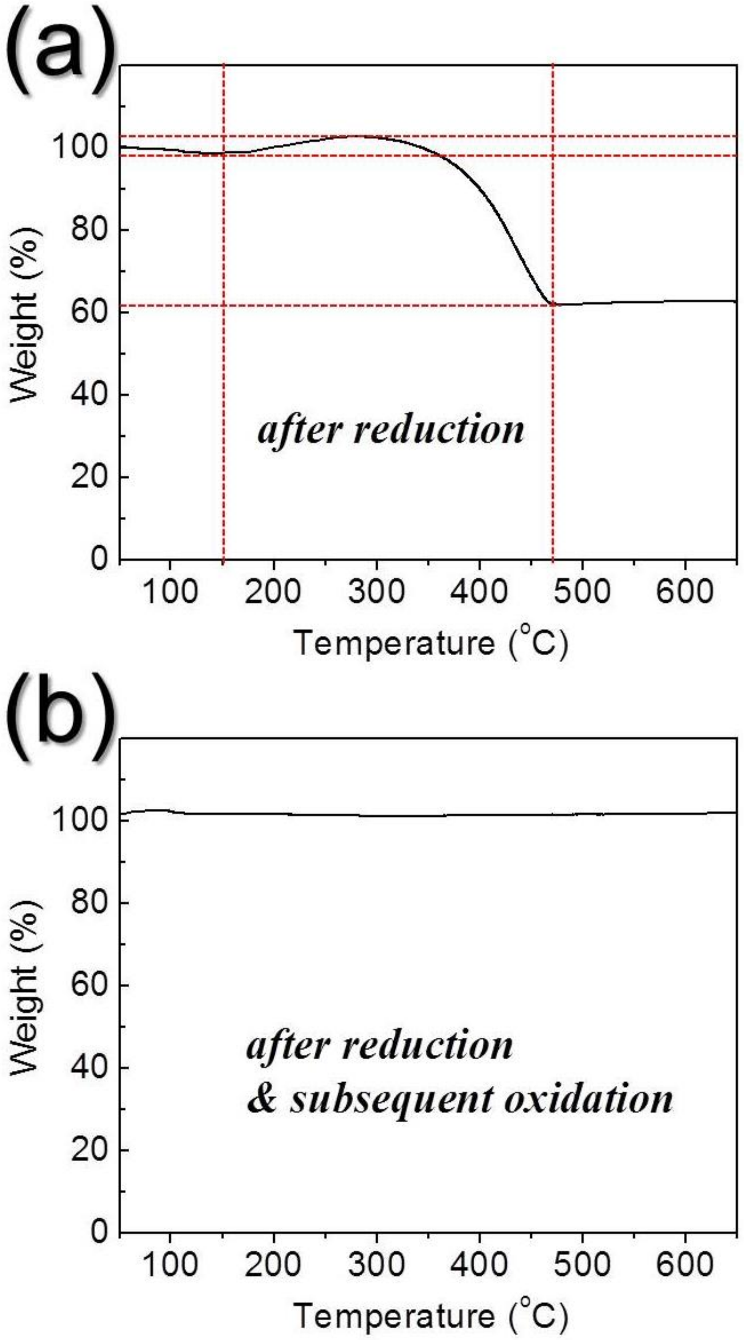

Figure S5 TG analyses of the (a) Sn-C composite powders obtained by reduction process at 300 ^oC under 10% H_2/Ar gas and (b) SnO_2 nanopowders with hollow structure obtained by oxidation of reduced Sn-C composite nanopowders at 500 ^oC under air.

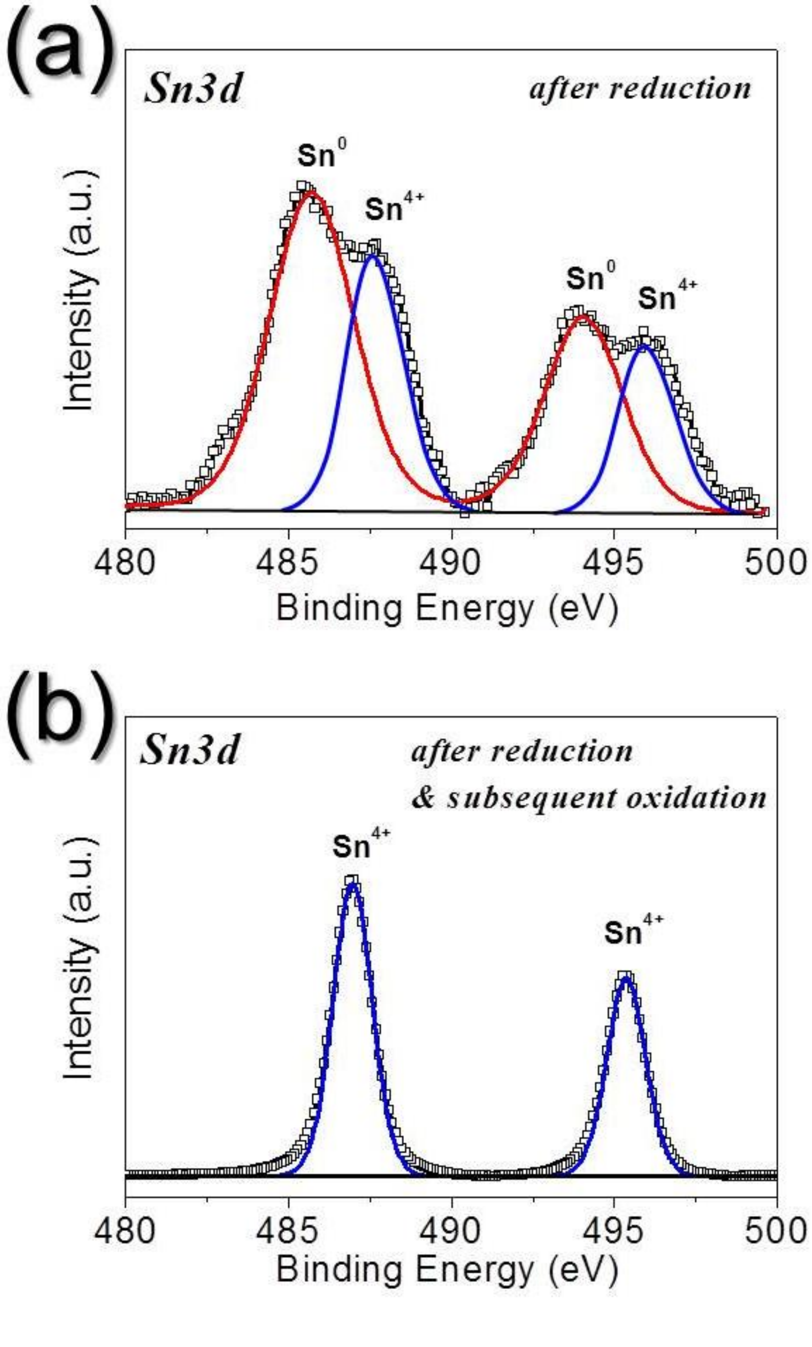

Figure S6 Sn3d XPS spectra of the (a) Sn-C composite powders obtained by reduction process at 300 ^{o}C under 10% H_2/Ar gas and (b) SnO_2 nanopowders with hollow structure obtained by oxidation of reduced Sn-C composite nanofibers at 500 ^{o}C under air.

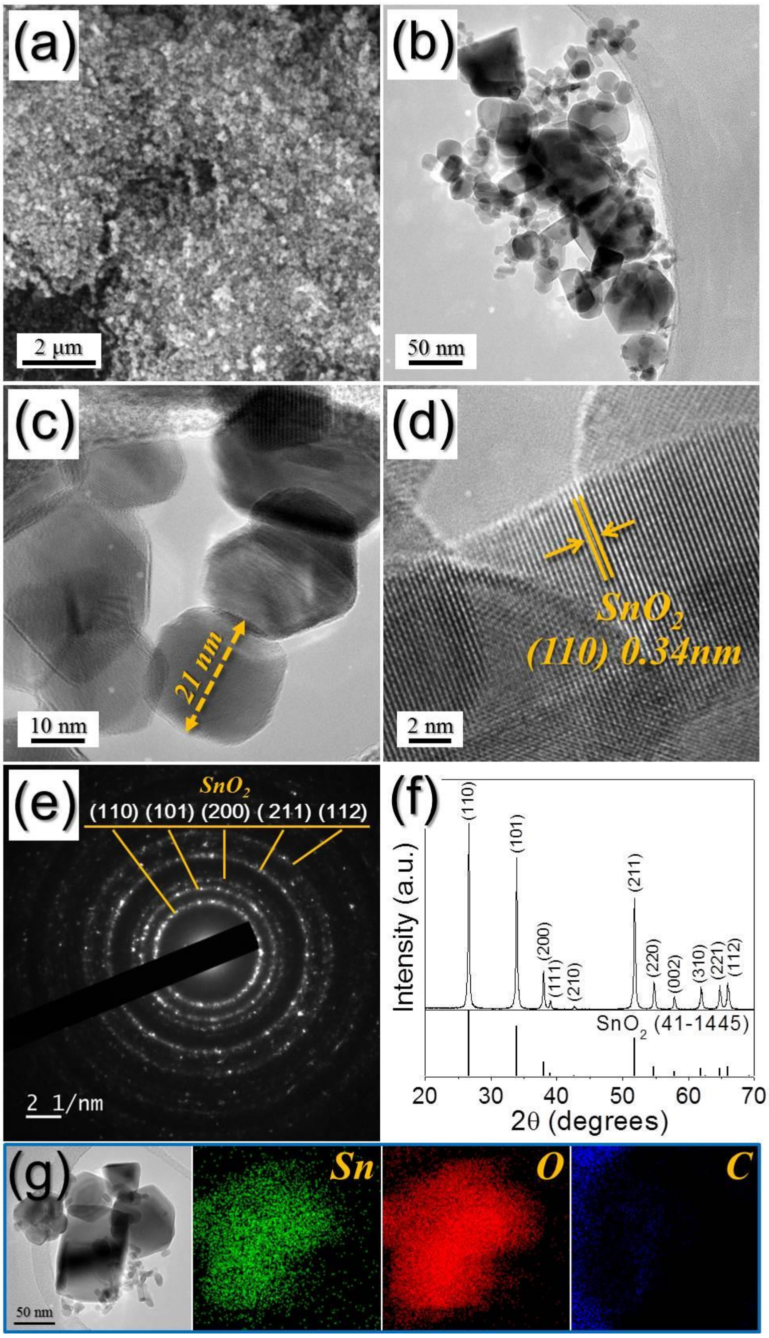

Figure S7 Morphologies, SAED pattern, XRD pattern, and elemental mapping images of the filled-structured SnO_2 nanoparticles formed by conventional flame spray pyrolysis process: (a) SEM, (b) and (c) TEM images, (d) HR-TEM image, (e) SAED pattern, (f) XRD pattern, and (g) elemental mapping images.

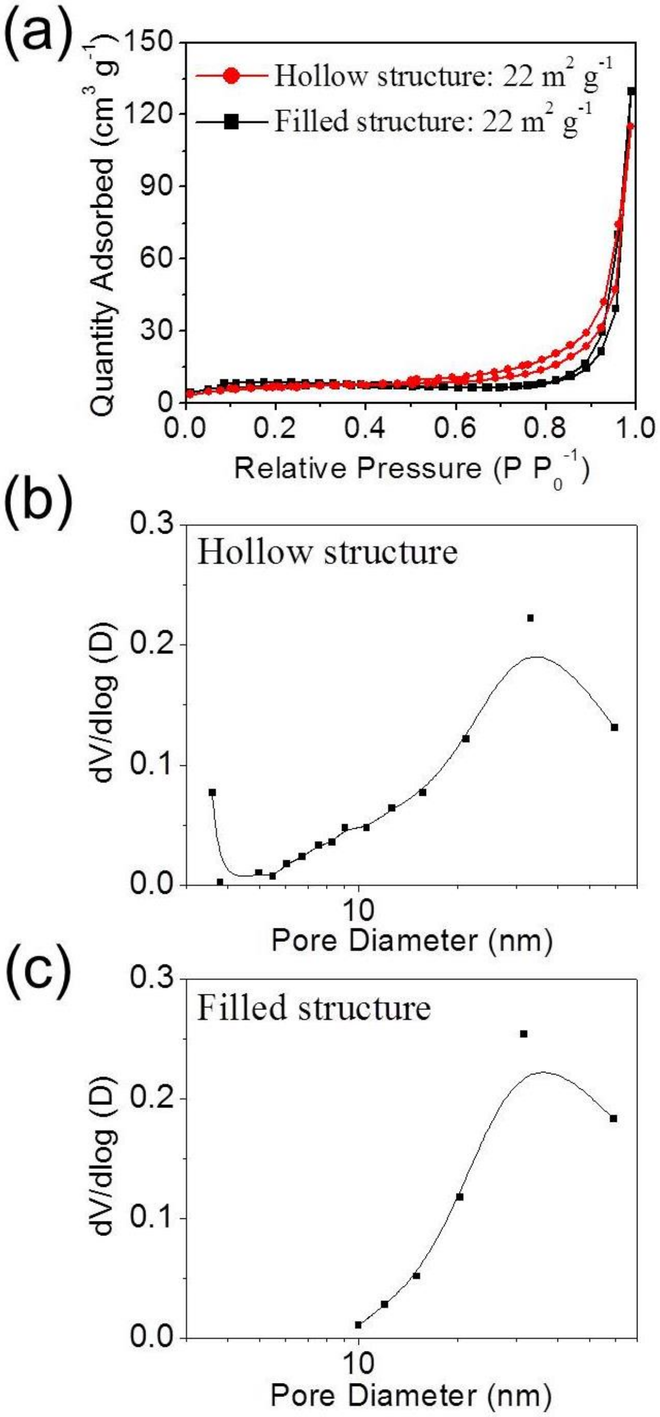

Figure S8 (a) N_2 adsorption-desorption isotherms (b-c) pore size distribution measured at 77 K for the SnO_2 hollow nanospheres formed by applying nanoscale Kirkendall diffusion process and filled-structured SnO_2 nanoparticles formed by conventional flame spray pyrolysis process.

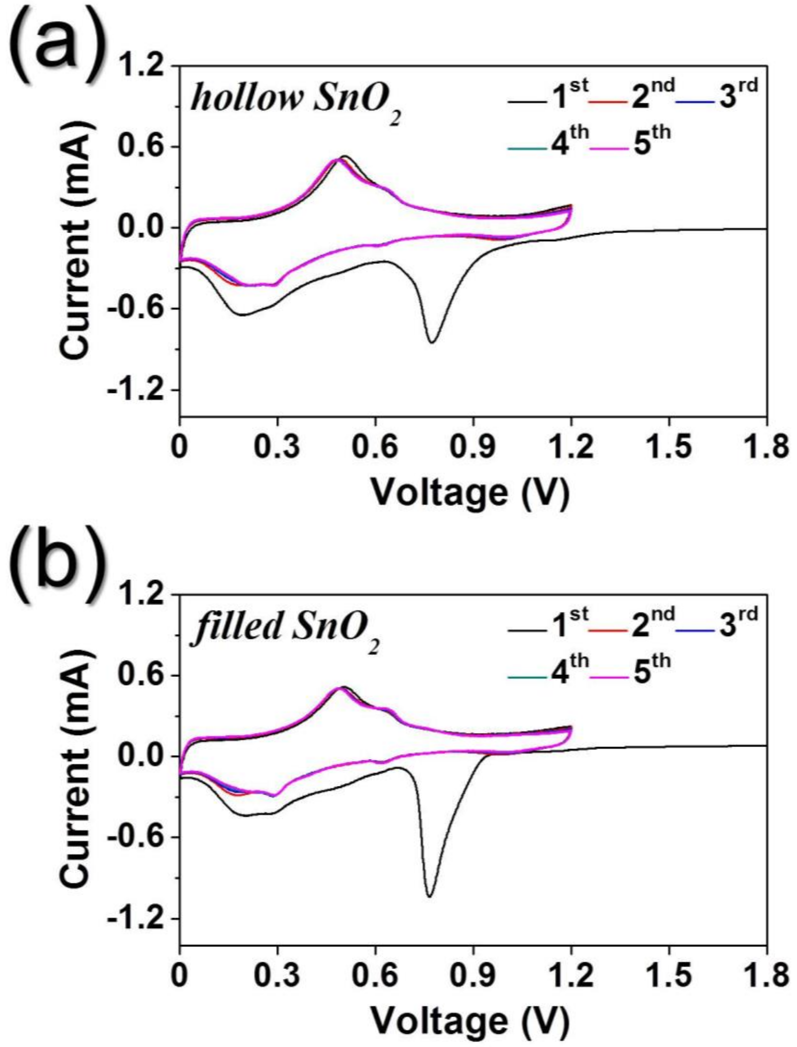

Figure S9 Cyclic voltammogram (CV) curves of (a) SnO_2 hollow nanospheres formed by applying nanoscale Kirkendall diffusion process and (b) filled-structured SnO_2 nanoparticles formed by conventional flame spray pyrolysis process for the first 5 cycles at a scan rate of 0.07 mV s^{-1}.

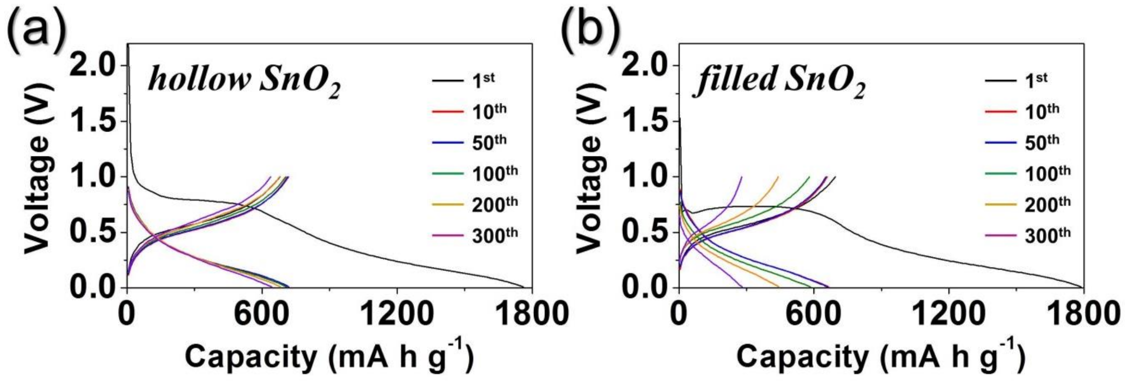

Figure S10 Discharge and charge voltage profiles of (a) SnO_2 hollow nanospheres formed by applying nanoscale Kirkendall diffusion process and (b) filled-structured SnO_2 nanoparticles formed by conventional flame spray pyrolysis process up to 300 cycles at a current density of 2.0 A g^{-1}.

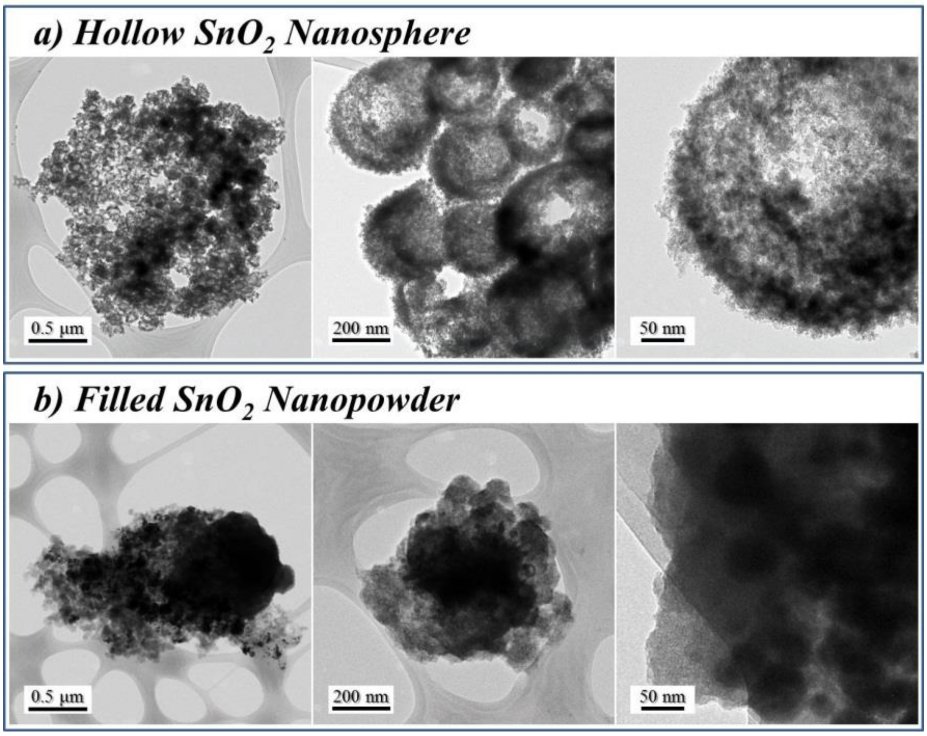

Figure S11 Morphologies of the SnO_2 nanopowders obtained after 300 cycles: (a) hollow SnO_2 nanospheres formed by applying nanoscale Kirkendall diffusion process and (b) filled structured SnO_2 nanopowders prepared by flame spray pyrolysis process.

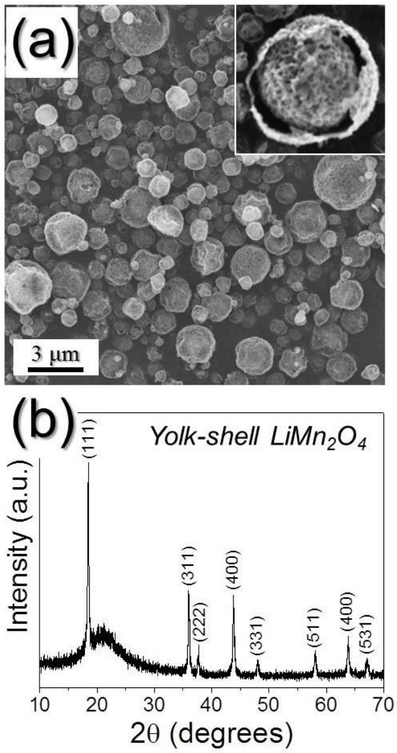

Figure S12 (a) SEM image and (b) XRD pattern of the yolk-shell structured $LiMn_2O_4$ powders prepared by spray pyrolysis process.

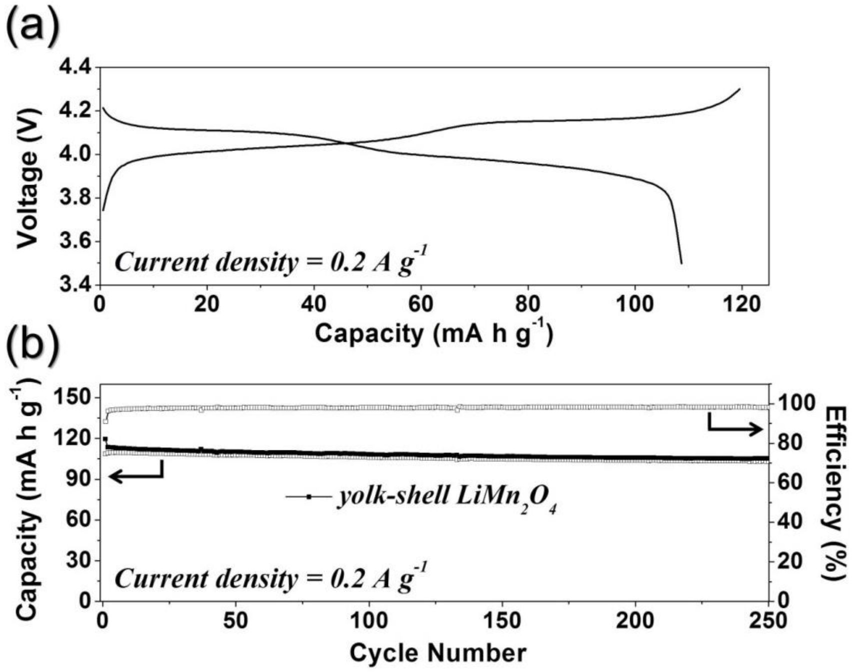

Figure S13 (a) Initial charge and discharge curves and (b) cycling performance of the yolk–shell $LiMn_2O_4$ powders at a current density of 0.2 A g^{-1}.

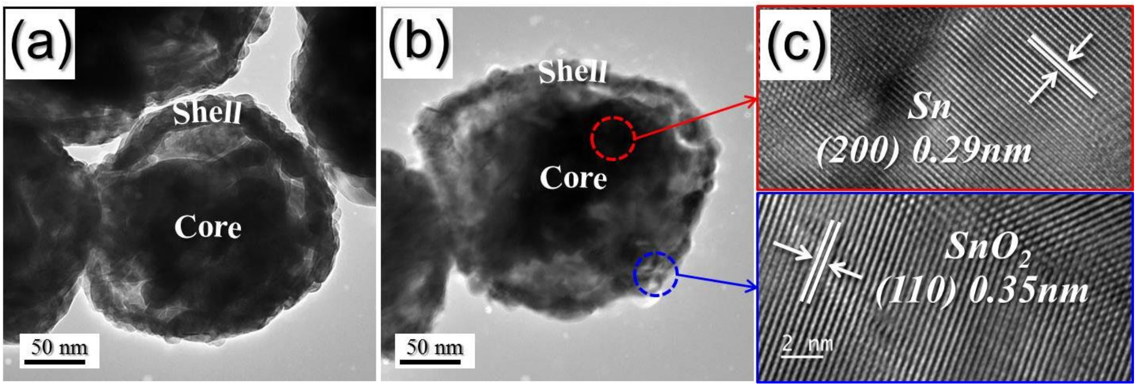

Figure S14 TEM images of the core-shell structured Sn@SnO_2 powder obtained after post-treatment of the Sn-C composite powders at 300 ^{o}C for 1h under air atmosphere.

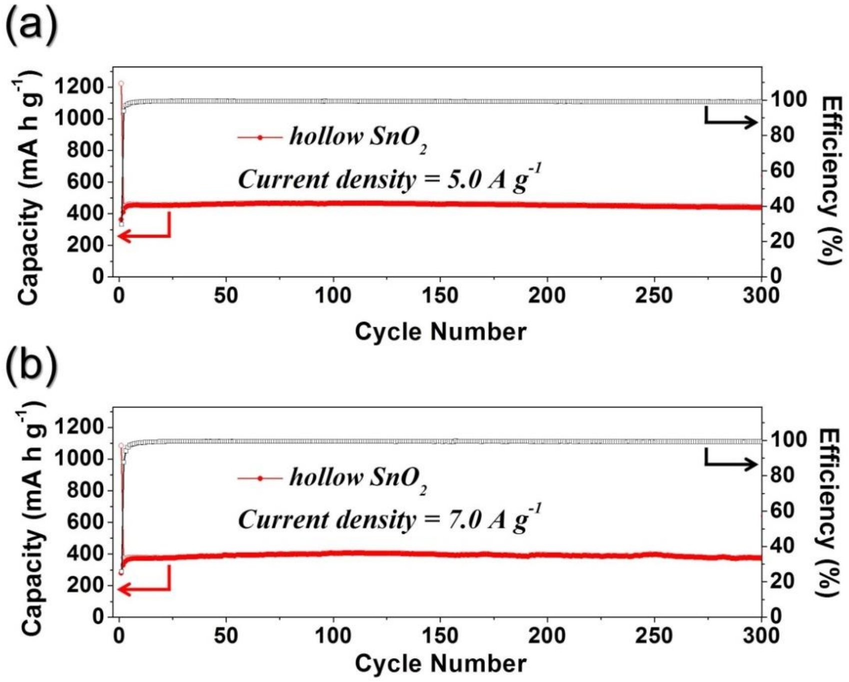

Figure S15 Cycling performances and Columbic efficiencies of SnO_2 hollow nanospheres formed by applying nanoscale Kirkendall diffusion process at different current densities: (a) 5.0 A g^{-1} and (b) 7.0 A g^{-1}.
